# Peptidomimetic Small Molecules Disrupt Type IV Secretion System Activity in Diverse Bacterial Pathogens

**DOI:** 10.1128/mBio.00221-16

**Published:** 2016-04-26

**Authors:** Carrie L. Shaffer, James A. D. Good, Santosh Kumar, K. Syam Krishnan, Jennifer A. Gaddy, John T. Loh, Joseph Chappell, Fredrik Almqvist, Timothy L. Cover, Maria Hadjifrangiskou

**Affiliations:** a Department of Pathology, Microbiology and Immunology, Vanderbilt University School of Medicine, Nashville, Tennessee, USA; b Department of Chemistry, Umeå University, Umeå, Sweden; c Umeå Centre for Microbial Research, Umeå University, Umeå, Sweden; d Department of Pharmaceutical Sciences, University of Kentucky, Lexington, Kentucky, USA; e Department of Medicine, Vanderbilt University School of Medicine, Nashville, Tennessee, USA; f Veterans Affairs Tennessee Valley Healthcare System, Nashville, Tennessee, USA

## Abstract

Bacteria utilize complex type IV secretion systems (T4SSs) to translocate diverse effector proteins or DNA into target cells. Despite the importance of T4SSs in bacterial pathogenesis, the mechanism by which these translocation machineries deliver cargo across the bacterial envelope remains poorly understood, and very few studies have investigated the use of synthetic molecules to disrupt T4SS-mediated transport. Here, we describe two synthetic small molecules (C10 and KSK85) that disrupt T4SS-dependent processes in multiple bacterial pathogens. *Helicobacter pylori* exploits a pilus appendage associated with the *cag* T4SS to inject an oncogenic effector protein (CagA) and peptidoglycan into gastric epithelial cells. In *H. pylori*, KSK85 impedes biogenesis of the pilus appendage associated with the *cag* T4SS, while C10 disrupts *cag* T4SS activity without perturbing pilus assembly. In addition to the effects in *H. pylori*, we demonstrate that these compounds disrupt interbacterial DNA transfer by conjugative T4SSs in *Escherichia coli* and impede *vir* T4SS-mediated DNA delivery by *Agrobacterium tumefaciens* in a plant model of infection. Of note, C10 effectively disarmed dissemination of a derepressed IncF plasmid into a recipient bacterial population, thus demonstrating the potential of these compounds in mitigating the spread of antibiotic resistance determinants driven by conjugation. To our knowledge, this study is the first report of synthetic small molecules that impair delivery of both effector protein and DNA cargos by diverse T4SSs.

## IMPORTANCE

Many human and plant pathogens utilize complex nanomachines called type IV secretion systems (T4SSs) to transport proteins and DNA to target cells. In addition to delivery of harmful effector proteins into target cells, T4SSs can disseminate genetic determinants that confer antibiotic resistance among bacterial populations. In this study, we sought to identify compounds that disrupt T4SS-mediated processes. Using the human gastric pathogen *H. pylori* as a model system, we identified and characterized two small molecules that prevent transfer of an oncogenic effector protein to host cells. We discovered that these small molecules also prevented the spread of antibiotic resistance plasmids in *E. coli* populations and diminished the transfer of tumor-inducing DNA from the plant pathogen *A. tumefaciens* to target cells. Thus, these compounds are versatile molecular tools that can be used to study and disarm these important bacterial machines.

## INTRODUCTION

Numerous bacterial species translocate effector molecules into target cells to subvert host defense mechanisms and hijack cellular processes. Delivery of bacterial effectors can be achieved using elaborate secretion systems that are assembled in response to specific environmental stimuli, such as direct bacterial contact with target cells (1). Type IV secretion systems (T4SSs) are extraordinarily versatile contact-dependent cargo delivery systems that are both phylogenetically and functionally diverse (2). These membrane-spanning systems are composed of conserved core complex subunits, as well as species-specific components that afford apparatus specialization and facilitate occupation of specific intracellular and extracellular niches (3–5).

T4SSs can be divided into three subfamilies: (i) DNA conjugation machines, (ii) DNA uptake/release systems that exchange DNA with the extracellular milieu, and (iii) effector translocator systems (2, 6, 7). T4SSs contribute to the pathogenesis of disease caused by several human pathogens, including *Brucella*, *Bartonella*, *Coxiella*, *Rickettsia*, *Legionella pneumophila*, *Helicobacter pylori*, and phytopathogens such as *Agrobacterium tumefaciens* (1, 3). Elegant studies of the prototypical *vir* T4SS effector translocator system in *A. tumefaciens* (4, 8, 9) have laid the groundwork for the study of T4SSs in other bacterial species, including the distantly related *cag* T4SS that is harbored by virulent strains of the gastric bacterium *H. pylori* (10–12).

*H. pylori* can persist within the human gastric mucosa, often for the lifetime of the individual. Although the majority of *H. pylori*-infected individuals remain asymptomatic, colonization by *H. pylori* is associated with a broad range of clinical outcomes ranging from non-atrophic gastritis to severe disorders including gastric and duodenal ulcers and gastric adenocarcinoma (13). These severe gastric diseases occur more frequently in individuals who are colonized by *H. pylori* strains that produce a T4SS encoded by the 40-kb *cag* pathogenicity island (*cag* PAI) (11). The *cag* T4SS translocates the oncogenic bacterial protein CagA (10, 12, 14), as well as peptidoglycan (15), directly into the cytoplasm of gastric epithelial cells. One consequence of *cag* T4SS activity is NF-κB activation (16, 17) and increased production of proinflammatory cytokines such as interleukin 8 (IL-8) (18). However, we lack a fundamental understanding of *cag* T4SS apparatus assembly, and the mechanisms of CagA translocation and peptidoglycan delivery remain unresolved (10–12, 14).

When in contact with human gastric epithelial cells, *H. pylori* produces filamentous structures at the bacterium-host cell interface. Formation of these structures requires several genes carried on the *cag* PAI (19–22). These structures are thought to be an extracellular portion of the *cag* T4SS, analogous to the F pilus in conjugative T4SSs, and thus, these *H. pylori* structures have been termed *cag* T4SS pili (19–21). The composition of *cag* T4SS pili is not well defined, but several *cag* PAI-encoded proteins have been reported to localize to the pilus (21–24). The effector protein CagA has been localized at or near the tips of *cag* T4SS pili (21, 24, 25), and *H. pylori* mutants that fail to produce pili are unable to translocate CagA (19, 20, 24, 25), indicating that *cag* T4SS pili have an important role in T4SS function. In contrast to the requirement of pili for the actions of the *H. pylori* *cag* T4SS, the delivery of *A. tumefaciens* oncogenic plasmid-derived transfer DNA (T-DNA) to target plant cells does not require biogenesis of the *vir* T4SS-associated T-pilus (8, 26, 27). Although the structures of related *Escherichia coli* conjugative T4SSs have been resolved (6, 7), the precise mechanism of DNA transport across the bacterial envelope remains elusive.

Chemical genetics enable the dissection of processes through the use of small-molecule inhibitors, which offer temporal control and reversibility (28). Thus far, there are very few chemical tools available for the study of T4SS-dependent processes in *H. pylori*. One study reported the discovery of salicylidene acylhydrazide inhibitors of the *Brucella* assembly factor VirB8, but the applicability of these compounds to other T4SSs has not been reported (29, 30). Other groups have reported identification of small molecules that inhibit the VirB11 ATPase of the *cag* T4SS (31, 32), but because these compounds are ATP mimetics that may inhibit other cellular ATPases, their utility for enhancing our understanding of *cag* T4SS apparatus assembly is limited.

We previously published a significant body of work describing the development of peptidomimetic small molecules (termed pilicides) that disrupted chaperone-usher pathway (CUP) pilus formation in *E. coli* (33–37). Structurally, these compounds contained a central ring-fused 2-pyridone peptidomimetic fragment (33, 34). Utilizing a compound library based around this scaffold, we performed a phenotypic screen for compounds that affected function or assembly of the *H. pylori* *cag* T4SS. We identified two closely related analogues, C10 and KSK85 that attenuated the delivery of peptidoglycan and CagA to host cells. We show that the T4SS targeting effects of C10 and KSK85 are not limited to *H. pylori*; C10 and KSK85 also inhibit interbacterial conjugative T4SS-mediated DNA transfer in *E. coli* and impair *vir* T4SS-dependent T-DNA delivery by *A. tumefaciens* in a plant model of infection. Moreover, we demonstrate that while KSK85 impairs formation of *H. pylori* *cag* T4SS-associated pili, C10 impairs T4SS-mediated transport without impacting T4SS pilus biogenesis. Thus, these small molecules can be used to dissect the assembly of T4SSs in divergent proteobacteria.

## RESULTS

### Identification of small molecules that disrupt *H. pylori cag* type IV effector delivery.

**FIG 1:**
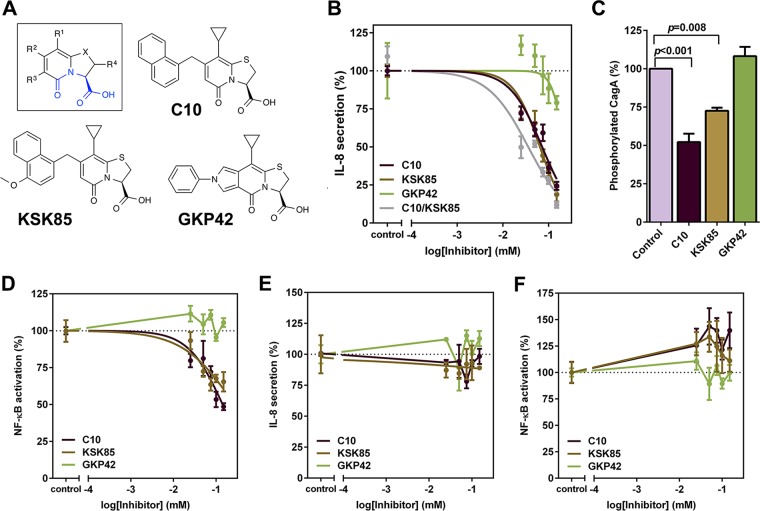
Peptidomimetic small molecules disrupt activity of the *H. pylori* *cag* T4SS. (A) Compounds evaluated in this study contain a common peptidomimetic ring-fused 2-pyridone backbone structure (shown in blue in the inset). (B and C) Effects of C10 and KSK85 on T4SS-dependent activation of IL-8 synthesis and secretion by cultured AGS gastric epithelial cells (B) and on CagA translocation into cultured human gastric epithelial cells (150 µM final compound concentrations assayed) (C). (C) Densitometry analysis of tyrosine-phosphorylated CagA normalized to total CagA in five independent biological replicates was performed by ImageJ analysis. (D) Effects of C10 and KSK85 on *H. pylori* T4SS-dependent NF-κB activation in AGS cells. (E and F) IL-8 secretion (E) and NF-κB activation (F) stimulated by recombinant human TNF-α (in the absence of *H. pylori*). Graphs of IL-8 secretion and NF-κB activation depict the means ± standard errors of the means (SEM) (error bars) of at least three biological replicate experiments. *P* values in panel C were calculated by one-way ANOVA. See also Fig. S1 in the supplemental material and Table 1.

A functionally active *cag* T4SS in *H. pylori* induces secretion of the proinflammatory cytokine IL-8 and activation of NF-κB signaling when *H. pylori* is co-cultured with gastric epithelial cells (16–18, 38). We screened a series of peptidomimetic small molecules (see Table S1 in the supplemental material) for their capacity to inhibit secretion of IL-8 and activation of NF-κB signaling in *H. pylori*-gastric epithelial cell culture (Fig. 1; see Fig. S1A in the supplemental material). This focused library consisted of 22 compounds from our in-house collection that contain a central peptidomimetic 2-pyridone motif (Table S1). In addition to compounds known to affect the assembly of type 1 pili or curli in *E. coli* (e.g., EC240 and FN075) (36, 37, 39), we included analogues prepared when developing synthetic methodologies (e.g., C10, MS218, and MS383) (40–42) and from natural-product-inspired diversity-oriented synthesis (e.g., MS542 and MS610) (43, 44). None of the compounds with known activity against CUP pilus assembly in *E. coli* affected *cag* T4SS activity at the concentrations tested. Two compounds significantly reduced *cag* T4SS-dependent IL-8 secretion and NF-κB activation (C10 and KSK85 [Table 1]) in a dose-dependent manner (Fig. 1B and D). The effects of C10 and KSK85 were additive when both compounds were present at equal concentrations in *H. pylori*-gastric epithelial cell co-cultures (Fig. 1B, gray line). We included an inactive tricyclic analogue GKP42 (Fig. 1A) as a non-inhibitory compound. GKP42 had minimal effects or no effect on IL-8 secretion at the highest concentrations tested (Fig. 1B) and did not attenuate NF-κB activation (Fig. 1D) or exhibit activity in subsequent assays*.* Exposure to C10, KSK85, or GKP42 did not impact viability of gastric epithelial or bacterial cells (Fig. S1B and S1C) at concentrations up to 150 µM.

**TABLE 1:** Fifty percent effective concentrations for the attenuation of *cag* T4SS-dependent IL-8 secretion by peptidomimetic small molecules^a^

Compound	Log EC_50_ (SEM) [95% CI]	Hill slope (SEM) [95% CI]	EC_50_ (µM) [95% CI]
C10	−1.19 (0.0322) [−1.26 to −1.12]	−1.49 (0.224) [−1.95 to −1.02]	64.5 [55.3−75.3]
KSK85	−1.14 (0.0337) [−1.21 to −1.07]	−1.23 (0.188) [−1.62 to −0.839]	72.2 [61.4−84.8]
C10/KSK85^b^	−1.45 (0.0694) [−1.59 to −1.30]	−0.98 (0.201) [−1.40 to −0.562]	35.8 [25.7−50]
GKP42	−0.648 (0.229) [−1.12 to −0.172]	−3.12 (3.21) [−9.80 to 3.56]	225 [75.2−673]

a Compounds were added to *H. pylori*-gastric epithelial cell monolayers for the duration of the coculture experiment (4.5 h), and IL-8 secretion was evaluated by anti-human IL-8 enzyme-linked immunosorbent assay (ELISA) as described in Text S1 in the supplemental material. Fifty percent effective concentrations (EC_50_s) were calculated from normalized IL-8 secretion levels, where IL-8 levels obtained from compound-treated samples were expressed as a percentage of values obtained in the vehicle control wells (0.3% DMSO, final concentration for all samples). Percentages from separate biological replicate samples were normalized to a range of 10 to 100%, where maximal T4SS inhibition is defined as 10% and 100% represents full T4SS activity. EC_50_ values were calculated in GraphPad using nonlinear regression of normalized IL-8 secretion values [variable slope four parameter, log(agonist) versus response]. The standard errors of the means (SEM) are shown in parentheses for log EC_50_ and Hill slope. The 95% confidence intervals (95% CI) are shown in brackets for log EC_50_, Hill slope, and EC_50_.

b C10 and KSK85 were added at equal concentrations ranging from 25 µM to 150 µM each.

The inhibition of *cag* T4SS-mediated IL-8 secretion and NF-κB activation by C10 and KSK85 prompted us to measure CagA delivery to gastric epithelial cells. CagA is phosphorylated at conserved tyrosine residues upon T4SS-dependent translocation into the host cell (12). Thus, translocated CagA can be detected by immunoblot analysis probing for the presence of phosphorylated CagA. Compared to vehicle-only and GKP42-treated controls, exposure to C10 and KSK85 significantly reduced the amount of tyrosine-phosphorylated CagA detected in cultured gastric epithelial cells (Fig. 1C; see Fig. S1E in the supplemental material). Compound treatment of AGS gastric epithelial cells in the absence of bacteria did not affect the ability of the cells to signal through the canonical tumor necrosis factor alpha (TNF-α) pathway, which leads to activation of NF-κB and subsequent synthesis and secretion of IL-8 (Fig. 1E and F). These compounds did not prevent bacterial adherence to gastric epithelial cells at concentrations ranging from 25 µM to 150 µM (Fig. S1D), indicating that the observed reduction in *cag* T4SS activity was not a consequence of fewer bacterial cells establishing contact with gastric epithelial cells. The compounds did not impair type V secretion system-mediated release of the cytotoxin VacA into cell culture supernatants (Fig. S1F), suggesting that C10 and KSK85 did not broadly impact bacterial secretory processes. C10 and KSK85 inhibited *cag* T4SS-mediated NF-κB activation by *H. pylori* strains of different phylogeographic origins (Fig. S1G and S1H). These data indicate that the observed compound effects were not strain specific. Further consideration of structure-activity relationships underlying the capacity of the compounds to inhibit *cag* T4SS function is presented in Text S1 in the supplemental material.

### KSK85 impairs T4SS-associated pilus formation.

**FIG 2:**
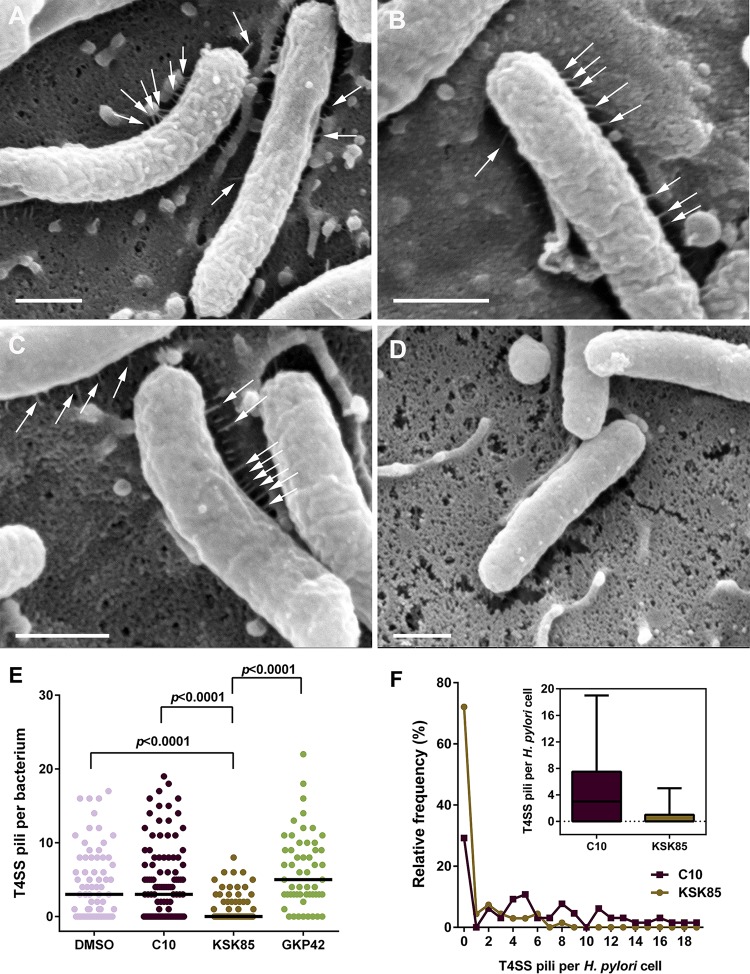
KSK85 inhibits assembly of T4SS pili at the bacterium-host cell interface. (A to D) Field-emission scanning electron microscopy (FESEM) evaluating the T4SS piliation state of *H. pylori* (white arrows) treated with vehicle (A), noninhibitory compound GKP42 (B), C10 (C), or KSK85 (D). Bars, 500 nm. (E) Enumeration of T4SS pili per bacterial cell. The short black lines in panel E represent the geometric mean of each distribution. (F) Proportion of *H. pylori* that elaborate T4SS pili in the presence of C10 and KSK85. The inset shows the median number of pili per bacterial cell (boxes) and the maximum number of T4SS pili observed per individual *H. pylori* cell (whiskers).

The peptidomimetic 2-pyridone scaffold used to generate C10 and KSK85 was designed to ablate pilus biogenesis in uropathogenic *E. coli* (35, 36). We therefore investigated the effects of C10 and KSK85 on the assembly of *cag* T4SS-associated pili at the bacterium-host cell interface. *H. pylori* exposed to C10, GKP42, or vehicle (Fig. 2A to C) produced similar numbers of *cag* T4SS pili, while treatment with KSK85 abrogated pilus formation in the majority of *H. pylori* cells (Fig. 2D to F). A small proportion of KSK85-treated *H. pylori* formed pili, which could account for the low levels of CagA translocation in KSK85-treated samples (Fig. 1C; see Fig. S1E in the supplemental material); however, the proportion of piliated cells, as well as the number of pili produced by each bacterial cell, was markedly reduced compared to C10-, GKP42- and vehicle-treated samples (Fig. 2E and F).

### CagA attenuates the disruptive effects of C10 and KSK85 on T4SS activity.

**FIG 3:**
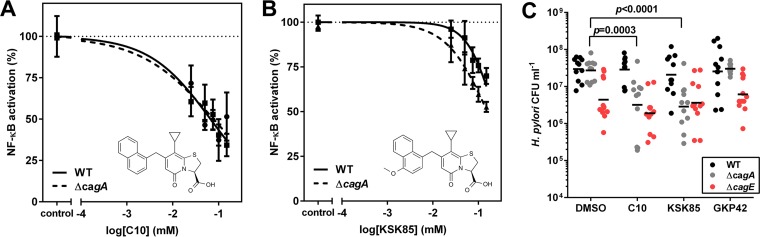
C10 and KSK85 disrupt *cag* T4SS activity in the absence of CagA. (A and B) T4SS-dependent NF-κB activation by WT *H. pylori* and *H. pylori* Δ*cagA* mutant in the presence of C10 (A) and KSK85 (B). The values are means ± standard errors of the means (error bars) derived from four biological replicate experiments. (C) CFU of adherent *H. pylori* on the surface of gastric epithelial cells at 6 h postinfection. The short black bars depict the geometric means of eight biological replicates. The *P* values in panel C were calculated by two-tailed Mann-Whitney test.

Activation of NF-κB signaling and induction of IL-8 secretion can occur in response to translocated CagA and/or peptidoglycan (16, 45). It is thus possible that C10 and KSK85 also influence the translocation of peptidoglycan or other unidentified effector molecules. To assess whether the inhibitory effects of the compounds on NF-κB signaling and IL-8 secretion were attributable to blocking one or both types of translocation, we evaluated the effects of KSK85 and C10 on an *H. pylori* Δ*cagA* mutant. The effects of C10 on *H. pylori* Δ*cagA* mutant were the same as those observed in the wild-type (WT) strain (Fig. 3A). These results suggest that the inhibitory effects of C10 on NF-κB activation are primarily attributable to blocking translocation of peptidoglycan or additional effector molecules (Fig. 3A). The ability of KSK85 to disrupt *cag* T4SS-mediated NF-κB activation was reduced compared to the inhibitory effects of C10 (Fig. 3A versus Fig. 3B); however, the inhibitory effects of KSK85 were significantly enhanced in the *cagA* mutant strain (*P* = 0.004 by paired *t* test) compared to the WT strain (Fig. 3B). To investigate the basis for this phenomenon, we first analyzed whether CagA is important for pilus assembly and observed that the Δ*cagA* mutant produced T4SS-associated pili at levels similar to those produced by the WT (see Fig. S2A in the supplemental material). Thus, although KSK85 inhibits formation of *cag* T4SS pili, loss of CagA does not preclude elaboration of pilus structures that are targeted by KSK85. In addition, the absence of CagA did not influence adherence of bacteria to the gastric epithelial surfaces (Fig. S2B), indicating that pilus-associated CagA does not facilitate binding of *H. pylori* to target host cells. Collectively, these data suggested that the inhibitory activity of KSK85 is reduced in the presence of CagA, potentially through CagA-compound interactions.

*H. pylori* exploits the surface of gastric epithelial cells as a replicative niche by using *cag* T4SS-dependent processes to control cell polarity (46). Therefore, we tested the effects of the compounds on replication of *H. pylori* co-cultured with gastric epithelial cells. In accordance with a previous report (46), fewer CFU of a Δ*cagE* mutant (deficient in an essential ATPase of the *cag* T4SS and defective in pilus production) were consistently recovered from AGS cell co-culture at 6 h post-infection compared to recovery of the WT strain, regardless of compound exposure (Fig. 3C). The Δ*cagE* mutant exhibited WT adherence properties (Fig. S2B), ruling out the possibility that the differential recovery of bacteria was attributable to differences in adherence. Similar numbers of CFU of the WT strain and *cagA* mutant were recovered from control co-cultures (treated with the vehicle dimethyl sulfoxide [DMSO]) (Fig. 3C). Interestingly, our studies revealed that the numbers of Δ*cagA* CFU recovered from co-culture at 6 h postinfection were reduced at least 10-fold in the compound-treated wells compared to the control DMSO-treated bacteria (Fig. 3C). This result indicated that loss of CagA enhances the T4SS inhibitory capacity of the compounds when *H. pylori* are in contact with host cells. However, we cannot rule out the possibility that the reduction in recovered Δ*cagA* CFU stems from altered host cell phenotypes that arise as a consequence of CagA translocation, which may confer survival benefits to the bacterium at this host-microbe interface.

### C10 and KSK85 significantly reduce T4SS-dependent DNA transfer in divergent proteobacteria.

**FIG 4:**
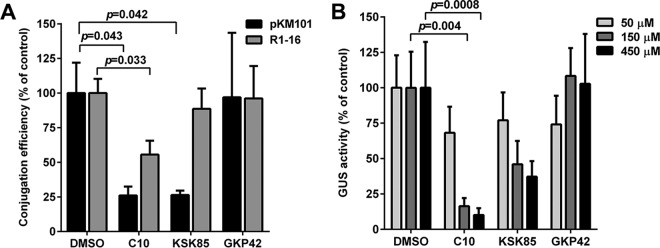
Peptidomimetic small molecules target diverse type IV secretion systems. (A) DNA conjugation efficiency by the pKM101 and R1-16 gene-encoded T4SSs in the presence of C10, KSK85, or GKP42 (150 µM assayed). Results represent mean conjugation efficiencies plus SEM for five independent experiments. (B) A fluorescence-based assay was used to quantify β-glucuronidase activity of *Nicotiana benthamiana* zones coinfiltrated with *A. tumefaciens* GV3101 pCAMBIA::GUS and DMSO, C10, KSK85, or GKP42 at the indicated concentrations. Values are means plus SEM of three biological replicates containing at least three leaves with multiple zones of agroinfiltration per leaf per biological replicate. *P* values were calculated by one-way ANOVA with Dunnett’s posthoc correction for multiple comparisons.

Given the incredible diversity of T4SS across phyla with respect to both function and apparatus architecture (1–3, 47), we investigated whether C10 and KSK85 attenuate T4SS processes in other bacterial species. We first analyzed the ability of C10 and KSK85 to prevent DNA transfer through the IncN group conjugative T4SS encoded by genes carried on pKM101 and the IncF group R1-16 conjugative T4SS in *E. coli* (7, 9). Compared to vehicle controls, C10 and KSK85 reduced the DNA conjugation efficiency of pKM101 in a statistically significant manner (Fig. 4A). Compound treatment did not impair *E. coli* growth (see Fig. S3A in the supplemental material), suggesting that the observed decrease in numbers of transconjugants was not due to reduced replication of the bacteria in the presence of compounds. Analysis of DNA conjugation efficiency of the derepressed R1-16 system (lacking a regulatory element that restricts DNA conjugation) demonstrated that C10 reduced DNA transfer, whereas KSK85 did not (Fig. 4A), suggesting that R1-16 derepression can partially compensate for the T4SS inhibitory effects of KSK85.

Finally, we analyzed the ability of C10 and KSK85 to ablate *Agrobacterium* *vir* T4SS-mediated DNA translocation into recipient plant cells using an intron-containing β-glucuronidase (GUS) gene reporter assay. Species of the genus *Agrobacterium* carry genes that encode type IV conjugation systems that facilitate the delivery of plasmid-derived transfer DNA (T-DNA) into target plant cells (1, 2); the T-DNA is subsequently integrated randomly into the plant cell genome. Conjugation-mediated transfer of T-DNA from *A. tumefaciens* to plants can be readily monitored using an agroinfiltration system developed for tobacco (48). This method relies on the infiltration of *A. tumefaciens* into the interstitial spaces of a leaf, followed by incubation to allow for T-DNA transfer, integration of T-DNA into the nuclear DNA of transformed plant cells, and T-DNA expression. In our assay, use of the intron-GUS expression cassette permits GUS enzyme production only by suitably transformed plant cells because *A. tumefaciens* lacks the splicing machinery necessary for removal of introns. Qualitative analysis of GUS activity can be performed by staining using X-Gluc (5-bromo-4-chloro-3-indolyl-β-d-glucuronic acid, cyclohexylammonium salt) as the substrate (see Fig. S4A in the supplemental material). Using a fluorescence-based assay that measures GUS enzymatic activity, we determined that T-DNA transfer and genomic integration could be reliably measured and quantified at 48 h post-infection.

Using this approach, we evaluated the ability of C10 and KSK85 to prevent *A. tumefaciens*-mediated transfer of the GUS intron-containing gene. Similar to results obtained using the *H. pylori* *cag* T4SS model and *E. coli* conjugation systems (Fig. 1 and 4A), C10 significantly reduced GUS production compared to either DMSO-treated or GKP42-treated controls (Fig. 4B). In contrast, KSK85 did not prevent T-DNA transfer, consistent with previous studies indicating that the *vir* pilus appendage is dispensable for T-DNA translocation (26). *A. tumefaciens* growth was not impaired in the presence of C10 or KSK85 at concentrations up to 450 µM (see Fig. S3B in the supplemental material). In parallel, we utilized a qualitative carrot disk tumor formation assay to evaluate the inhibitory effects of the compounds on *A. tumefaciens* *vir* T4SS activity (49). We found that carrots impregnated with 150 µM C10 prior to inoculation with *A. tumefaciens* developed markedly fewer tumors compared to carrots treated with KSK85 and vehicle controls (Fig. S4B). Although the carrot disk assay does not allow for an evaluation of the half-lives of compounds during the period of carrot disk incubation or an assessment of the viability of *A. tumefaciens* infiltrated within carrot disks over the course of tumor formation, the effects of C10 in preventing tumor formation mirrored the decreased levels of T-DNA transfer measured in C10-treated leaves in our tobacco plant infection model. Collectively, our studies identified two compounds, C10 and KSK85, that exhibit inhibitory activity toward T4SS function in diverse bacteria.

## DISCUSSION

Type IV secretion systems are incredibly diverse nanomachines that vary in function and complexity across bacterial phyla (1, 2). The versatility of T4SS function confers a range of fitness advantages that significantly contribute to bacterial genome plasticity, pathogenic potential, and bacterial survival within distinct environments (2). Thus far, there have been relatively few studies aimed at developing inhibitors of T4SSs (29–32, 50), and previous studies largely relied on high-throughput screening methodologies to target one specific T4SS component. The intrinsic complexity of these multicomponent systems and the lack of detailed understanding of *cag* T4SS biogenesis mechanisms in *H. pylori* favor the use of a phenotypic screen to identify potential small-molecule modulators. Here, we identified two compounds from our focused peptidomimetic library that interfere with T4SS-dependent delivery of both protein and DNA cargo in diverse proteobacteria.

Understanding the process of the *H. pylori* *cag* T4SS apparatus assembly will undoubtedly require many years of investigation; utilizing chemical probes such as C10 and KSK85 will greatly augment current efforts. For example, our field-emission scanning electron microscopy (FESEM) studies indicated that while C10 does not prevent assembly of the *cag* T4SS-associated pilus, KSK85 significantly inhibits T4SS pilus biogenesis (Fig. 2). Remarkably, these compounds differ by a single methoxy group on the naphthyl substituent of the 2-pyridone ring (Fig. 1A). Both compounds reduced *cag* T4SS-dependent IL-8 secretion and NF-κB activation by WT *H. pylori* (Fig. 1), yet KSK85 exhibited greater inhibitory disruption of T4SS activity in a Δ*cagA* mutant (Fig. 3). Further studies are under way to dissect the basis for functional differences between the two compounds. Moreover, the identified compounds were modestly effective at reducing T4SS-related activity (Table 1), and thus, they can serve as prototypical scaffolds for further development of compounds that target *cag* T4SS activity.

The observation that only a small subset of individuals colonized by *H. pylori* develop severe gastric disease is reflective of a finely regulated host-microbe coevolution spanning many thousands of years (13, 51, 52). Injection of CagA into gastric epithelial cells likely provides several colonization advantages to *H. pylori*. The role of CagA as a eukaryotic signaling mimetic with the capacity to interact with multiple intracellular host cell components signifies a specialized function of this bacterial effector and highlights the integral contribution of the *cag* T4SS delivery machinery in mediating pathogenesis (11, 53, 54). A recent study described how translocated CagA allows *H. pylori* to usurp the surface of gastric epithelial cells as a replicative niche (46). Using C10 and KSK85 as molecular probes, we provide additional evidence that T4SS-dependent processes are important for *H. pylori* viability on the surfaces of gastric epithelial cells *in vitro* (Fig. 3C). Thus, these results highlight the utility of these chemical probes in dissecting pathogenicity determinants that contribute to bacterial colonization of the host.

When studying the effects of KSK85 and C10 on *E. coli* conjugation efficiency and on the *vir* T4SS harbored by *A. tumefaciens*, we observed that compared to KSK85, C10 was a more potent inhibitor of DNA transfer (Fig. 4A and B). There are multiple possible explanations for this observation. One possibility, given that previous studies have shown that the *vir* pilus appendage is not essential for DNA translocation into the plant cell (26, 27) is that KSK85 preferentially inhibits T4SSs that require the elaboration of a pilus to translocate their cargo. Conversely, since C10 is a more potent inhibitor of type IV secretion in a broad range of bacterial species, we hypothesize that C10 impacts a process independent of T4SS pilus biogenesis that is essential for T4SS cargo transport. In future investigations, it will be important to explore how these small molecule inhibitors may alter the synthesis or accumulation of T4SS protein components during apparatus biogenesis.

While we identified these small molecules as chemical probes to interrogate *H. pylori* *cag* T4SS biogenesis, our findings provide a foundation for the potential development of *H. pylori*-specific anti-virulence strategies (Fig. 1, Table 1, and Fig. 3). The *cag* T4SS harbored by *H. pylori* is one of the most significant virulence determinants associated with development of severe gastric disease (53, 55, 56). CagA, the only identified effector protein known to be injected into host gastric epithelial cells by *H. pylori*, is a bona fide oncoprotein that hijacks host cell signaling pathways to promote carcinogenesis (11, 53, 56, 57). The development of compounds that disarm *cag* T4SS-mediated processes may lead to new strategies for mitigating the threat associated with *H. pylori*-induced carcinogenesis in populations at high risk for developing gastric cancers (13). In this study, we identified 2-pyridone compounds that interfere with CagA translocation (Fig. 1C) and additionally block *cag* T4SS activity in a CagA-independent manner (Fig. 3). Interestingly, the efficacy of KSK85 against the *cag* T4SS was enhanced in a CagA mutant strain, which raises the possibility that KSK85 can interact with this oncoprotein. We speculate that the highly abundant CagA protein can serve as a protein sink within *H. pylori*, thereby sequestering compounds such as KSK85 away from cellular targets, leading to attenuation of T4SS inhibitory effects. We propose that CagA interacts with KSK85 to titrate the compound away from other cellular targets that are involved in T4SS function, while also preventing biogenesis of *cag* T4SS-associated pili in a CagA-independent manner. These compounds could potentially segregate CagA to bacterial compartments and serve as potential therapeutics; we are currently investigating the underlying mechanism for this observation.

The explosive emergence of antibiotic resistance among clinically significant bacterial populations highlights the critical role of T4SSs in the rapid propagation and exchange of mobile genetic elements (2). Resistance to current therapies continually arises due to selective pressure for bacteria to circumvent the bactericidal actions of antibiotics, and this poses a serious threat to public health. Rather than targeting essential cellular functions that ultimately kill the bacterium, utilizing compounds that reprogram and/or abort processes such as T4SS-mediated genetic exchange represents an attractive new avenue for drug development. Likewise, compounds that specifically target DNA conjugation would critically impact the rate and frequency of horizontal gene transfer in “hot spots” for this phenomenon (50), such as the gastrointestinal tract, without significantly altering the gross ecology of the host microflora. In summary, we present two structurally related compounds that impact T4SS-mediated processes involved in virulence factor secretion and dissemination of plasmid-borne antibiotic resistance markers. Thus, these compounds are excellent molecular scaffolds that can be exploited in the development of versatile chemical tools to dissect and disarm these important nanomachines.

## MATERIALS AND METHODS

### Synthesis of ring-fused 2-pyridones.

Complete synthetic methods and schemes are presented in Text S1 in the supplemental materials.

### Bacterial strains and growth conditions.

*H. pylori* strains 26695, G27, 98-10, and isogenic 26695 mutants (19, 20) were grown on Trypticase soy agar supplemented with 5% sheep blood (BD Biosciences) or brucella broth supplemented with 5% fetal bovine serum (FBS) at 37°C in 5% atmosphere CO_2_. *E. coli* MG1655 harboring the conjugative plasmid pKM101, *E. coli* MS411 harboring the R1-16 conjugative plasmid, and *E. coli* WM1652 recipient cells were grown on lysogeny broth (LB) agar or broth supplemented with ampicillin (50 µg/ml), kanamycin (50 µg/ml), or tetracycline (20 µg/ml), respectively. *Agrobacterium tumefaciens* C58 and its derivative GV3101 were maintained on LB plates supplemented with rifampin (25 µg/ml) at 28°C. *A. tumefaciens* GV3101 harboring pCAMBIA vectors were maintained on LB containing rifampin (25 µg/ml) and kanamycin (100 µg/ml).

### Cell culture.

AGS human gastric epithelial cells and the AGS NF-κB luciferase reporter cell line (21) were grown in 5% CO_2_ in RPMI 1640 medium supplemented with 10% FBS, 2 mM l-glutamine, and 10 mM HEPES buffer (complete RPMI).

### Cell viability assays.

*H. pylori*, *A. tumefaciens*, *E. coli*, or human cell lines were grown in complete RPMI or brucella broth supplemented with the indicated compounds or dimethyl sulfoxide (DMSO) for 6 to 24 h. Cell viability was assessed using CellTiter-Glo (Promega) according to the manufacturer’s protocol.

### IL-8 induction assays.

Quantitation of IL-8 secretion by gastric epithelial cells in contact with *H. pylori* was performed as previously described (20) and in Text S1 in the supplemental material.

### Quantitation of NF-κB signaling activation.

AGS cells stably transfected with an NF-κB-luciferase reporter (AGS NF-κB-luc) (21) were cultured for 24 h and treated as described for the IL-8 induction assays. After 4 h of coculture with *H. pylori*, cell monolayers were washed with sterile phosphate-buffered saline (PBS), and cell monolayer extracts were obtained by passive cell lysis. NF-κB-luciferase activity was measured by the Steady-Glo luciferase assay system (Promega) on a BioTek Synergy 4 plate reader, and plates were normalized to the DMSO vehicle control for each dilution series. AGS NF-κB-luc cells exposed to compounds in the absence of *H. pylori* were stimulated for 1 h with 10 ng/ml TNF-α and assayed by the Steady-Glo system to validate that compounds do not impact NF-κB signaling.

### CagA translocation assay.

Translocation of CagA into AGS cells was analyzed as described in reference 20 and Text S1 in the supplemental material.

### Scanning electron microscopy.

Overnight cultures of *H. pylori* were diluted to an optical density at 600 nm (OD_600_) of ~0.3 and incubated with shaking at 37°C and 5% CO_2_ for 1 h in the presence of vehicle or 150 µM compound and cocultured with AGS cells on tissue culture-treated coverslips (BD Biosciences) at a multiplicity of infection (MOI) of 100 in the presence of 150 µM compound or vehicle. *H. pylori*-AGS cell co-cultures were processed and imaged as described in references 19, 20, and 21 and Text S1 in the supplemental material.

### *H. pylori* viability in AGS cell coculture.

*H. pylori* pretreated with compound were added to AGS cells at an MOI of 100 in the presence of 150 µM compound and treated as described for IL-8 induction studies. After 6 h of coculture, RPMI 1640 medium was aspirated, and the wells were washed five times with sterile PBS to remove non-adherent bacteria. AGS cells with adherent *H. pylori* were dislodged in sterile PBS, and serial dilutions were plated to determine the number of *H. pylori* CFU. *H. pylori* survival experiments were performed at least three times with four replicate wells per experimental condition.

### Analysis of DNA conjugation efficiency.

Overnight cultures of donor *E. coli* MG1655 harboring pKM101, donor *E. coli* MS411 harboring R1-16, and recipient *E. coli* MG1655 (WM1652) bacteria were grown at 37°C with shaking at 225 rpm in LB plus antibiotic, diluted in antibiotic-free LB containing DMSO or 150 µM compound, and incubated with shaking at 37°C to reach an OD_600_ of ~0.3. Donor and recipient cells were mixed at a ratio of 1:1 on 0.22-µm-pore-size mixed-cellulose-ester filters (Millipore) on antibiotic-free LB plates for 2 h at 37°C. Filters were aseptically removed from LB plates and incubated in 100 µl antibiotic-free LB to dislodge *E. coli* from membranes. Serial dilutions were plated on the appropriate antibiotic (or dual-antibiotic) LB plates to determine the number of donor, recipient, and transconjugant cells. Colonies were enumerated after 24 h, and conjugation efficiencies were calculated by dividing the average number of transconjugates by the average number of donors (either pKM101 or R1-16 donor cells). Conjugation efficiency in the presence of compound is expressed as a percentage of conjugation efficiency in DMSO. Data represent six individual experiments for compounds C10 and KSK85 and three individual experiments for GKP42.

### Agroinfiltration and GUS assays.

Agroinfiltration and β-glucuronidase (GUS) fluorometric assays were performed as previously described (58, 59). Extensive experimental protocols are presented in Text S1 in the supplemental material.

### Carrot disk tumor formation assay.

Whole carrots were sterilized by soaking in 20% bleach for 30 min and subsequently sliced into disks of approximately 8 to 10 mm. The apical surface of each disk was placed on water agar (1.5%) medium containing no additional nutritional supplementation. Carrot disks were incubated with 100 µl of either DMSO, C10, KSK85, or GKP42 (final concentration of all compounds at 150 µM) in sterile PBS for 1 h to allow for the uptake of compounds into the carrot tissue. Replicate carrot disks were inoculated with 20 µl *Agrobacterium tumefaciens* C58 harvested from logarithmic-phase growth (OD_600_ of ~0.8) that had been pretreated for 1 h with shaking at 28°C in LB containing DMSO, C10, KSK85, or GKP42 (each compound at a final concentration of 150 µM). The plates were sealed with Parafilm and stored in the dark at room temperature for 3 weeks. Carrot tumors were photographed at 21 days postinoculation. Images are representative of at least five individual carrot disks.

### Statistical analysis.

Statistical analyses were performed using GraphPad Prism 6. Data are expressed as the mean ± standard error. Data comparisons from more than two groups were analyzed by analysis of variance (ANOVA) followed by Dunnett’s posthoc test for multiple comparisons against a single control (vehicle-treated samples).

## SUPPLEMENTAL MATERIAL

Text S1  Supplemental synthetic methods, supplemental synthetic schemes, supplemental structure-activity relationship analysis, and supplemental Materials and Methods Download Text S1, DOCX file, 0.1 MB

Figure S1  Effects of peptidomimetic small molecules on cell viability and T4SS-independent processes. (A) Schematic depicting the workflow of compound screening. A collection of ring-fused 2-pyridone compounds was initially evaluated at final concentrations of 150 µM for impact on host cell or *H. pylori* viability. Compounds that did not negatively affect either host or bacterial cell viability were subsequently evaluated for inhibition of *H. pylori* *cag* T4SS-dependent processes, including disruption of CagA translocation, induction of IL-8 secretion by cultured gastric epithelial cells, and NF-κB activation. (B and C) AGS (B) and *H. pylori* (C) cell viability as determined by the level of cellular ATP content after 6 h exposure to compounds. (D) Total *H. pylori* adherence to gastric epithelial cell monolayers in the presence of ring-fused 2-pyridones (final concentrations of 150 µM for all assayed compounds).Values in panels B to D represent the means plus SEM (error bars) of at least three biological replicates. (E) Representative image depicting CagA translocation into cultured gastric epithelial cells (tyrosine-phosphorylated CagA, immunoblotting with anti-PY99 antibody [IB: α-PY99]) versus levels of total CagA (IB: α-CagA). (F) Immunoblot depicting the relative amounts of *H. pylori* VacA secreted into cell culture supernatants when bacteria were grown in the presence of compounds or DMSO. (G and H) Effects of C10 (G) and KSK85 (H) on *cag* T4SS-dependent activation of NF-κB in an AGS reporter cell line by multiple *H. pylori* strains. Data points in panels G and H depict the means ± SEM (error bars) of at least three biological replicate experiments. Download Figure S1, TIF file, 1.3 MB

Figure S2  CagA is not required for T4SS pilus production or adherence to gastric epithelial cells. (A) Scanning electron microscopy analysis of T4SS pilus assembly by *H. pylori* Δ*cagA* mutant. Bar, 1 µm. (B) Total bacterial adherence of WT, Δ*cagA*, and Δ*cagE* strains to gastric epithelial cells in the presence of compounds. Bars represent the adherence of each strain normalized to DMSO-treated samples (mean plus standard error) and are representative of at least two biological replicate experiments. Download Figure S2, TIF file, 4.1 MB

Figure S3  Effects of peptidomimetic compounds on *E. coli* and *A. tumefaciens* viability. (A) Optical density measurements of the growth of *E. coli* MG1655 harboring pKM101 in the presence of vehicle, C10, KSK85, or GKP42 measured at 1 h intervals. (B) *A. tumefaciens* cell viability as determined by the level of cellular ATP content after 24 h growth in the presence of compounds at the indicated concentrations. Data points in panel A represent the mean OD_600_ of six independent samples. Data in panel B represent the mean cellular ATP content ± SEM compared to DMSO vehicle control samples and are representative of two biological replicate experiments. Download Figure S3, TIF file, 5.4 MB

Figure S4  Qualitative assessment of *A. tumefaciens* *vir* T4SS-dependent phenotypes. (A) Qualitative representation of T-DNA incorporation and GUS expression in tobacco leaves. Young, expanding leaves of *Nicotiana benthamiana* were infiltrated with *A. tumefaciens* GV3101 or *A. tumefaciens* GV3101 harboring pCAMBIA 1305.2 intronic GUS reporter gene expression cassette (pCAMBIA::GUS). A representative image of an *N*. *benthamiana* leaf stained histochemically for GUS enzyme activity demonstrating negative (GV3101) and positive (GV3101 pCAMBIA::GUS) incorporation of the β-glucuronidase reporter gene into *N*. *benthamiana* nuclear DNA is shown. (B) Qualitative carrot disk tumor assay demonstrating marked reduction of *A. tumefaciens* C58-induced tumors after a single administration (150 µM) of compound or equivalent volume of DMSO. Download Figure S4, TIF file, 3.6 MB

Table S1  Composition of peptidomimetic 2-pyridone focused screening library. The compounds (150 µM) were screened for phenotypic disruption of *cag* T4SS activity as measured by a significant decrease in IL-8 secretion or a significant decrease in NF-κB activation induced by WT *H*. *pylori* in co-culture with AGS gastric epithelial cells. The compounds were determined to be toxic to either AGS cells or *H*. *pylori* by a significant decrease in cellular ATP at 18 h of incubation. Table S1, DOCX file, 0.3 MB
