# Supplementary material for: Peptidomimetic Small Molecules Disrupt Type IV Secretion System Activity in Diverse Bacterial Pathogens
Source: mBio. 2016 Apr 26;7(2):e00221-16. doi: 10.1128/mBio.00221-16 (PMC4850256; doi:10.1128/mBio.00221-16)
Supplement: Text S1 — Supplemental synthetic methods, supplemental synthetic schemes, supplemental structure-activity relationship analysis, and supplemental Materials and Methods Download [file mbo002162780s1.docx]

**SUPPLEMENTAL MATERIAL**

**Peptidomimetic small molecules disrupt type IV secretion system activity in diverse bacterial pathogens**

**Carrie L. Shaffer^1^**, James A. D. Good^2,3^, Santosh Kumar^4^, K. Syam Krishnan^2^, Jennifer A. Gaddy^5,6^, John T. Loh^5^, Joseph Chappell^4^, Fredrik Almqvist^2,3^, Timothy L. Cover^1,5,6^, Maria Hadjifrangiskou^1*^

^1^Department of Pathology, Microbiology and Immunology, Vanderbilt University School of Medicine, Nashville, TN 37232

^2^Department of Chemistry, Umeå University, 901 87 Umeå Sweden

^3^Umeå Centre for Microbial Research, Umeå University, 901 87 Umeå, Sweden

^4^Department of Pharmaceutical Sciences, University of Kentucky, Lexington, KY 40536-0596

^5^Department of Medicine, Vanderbilt University School of Medicine, Nashville, TN 37232

^6^Veterans Affairs Tennessee Valley Healthcare System, Nashville, TN 37212

*Corresponding author, please send correspondence to [maria.hadjifrangiskou@vanderbilt.edu](mailto:maria.hadjifrangiskou@vanderbilt.edu)

**SUPPLEMENTAL Synthetic METHODS**

**General Synthetic Methods**

All reagents and solvents were used as received from commercial suppliers, unless indicated otherwise. Triethylamine was passed through activated alumina oxide and dried over 3Å molecular sieves prior to use. Microwave reactions were performed using a Biotage Initiator microwave synthesizer in sealed vessels with temperature monitoring by an internal IR probe. TLC was performed on aluminum backed silica gel plates (median pore size 60Å) and detected with UV light at 254 nm. Column chromatography was performed using silica gel with average particle diameter 50 µM (range 40-65 µM, pore diameter 53Å) and eluents are given in brackets. HPLC purifications were performed on a system equipped with a 250 × 21.5 mm Nucleodur® C18 HTEC (particle size 5 µM) semi-preparatory column using a flow rate of 20 mL/min and detection at 220 nm. Optical rotation was measured with a polarimeter at 25 °C at 589 nm. ^1^H and ^13^C NMR spectra were recorded on a 400 or 600 MHz spectrometer at 298 K and calibrated by using the residual peak of the solvent as the internal standard (CDCl_3_: δ_H_ = 7.26 ppm; δ_C_ = 77.16 ppm; CD_3_OD: δ_H_ = 3.31 ppm; δ_C_ = 49.00 ppm; DMSO-d_6_: δ_H_ = 2.50 ppm; δ_C_ = 39.50 ppm). HRMS was performed using a mass spectrometer with ESI-TOF (ESI+) with sodium formate used as the calibration chemical. Compounds are named according to IUPAC nomenclature by ACD ChemSketch 12.01 (Windows, Advanced Chemistry Development, Toronto, Canada). C10 was prepared as described previously (1). The chloromethyl intermediate **S3** (**Scheme S1**) and the methyl ester **S5** (**Scheme S2**) were prepared as described previously (2). 1-Bromo-4-methoxynaphthalene (**S1**) was prepared according to published procedures (3).

**Synthetic procedures and characterization of compounds**

**2-(4-Methoxy-1-naphthyl)-4,4,5,5-tetramethyl-1,3,2-dioxaborolane (S2).** 4,4,5,5-Tetramethyl-1,3,2-dioxaborolane (1.92 mL, 15.00 mmol) was added dropwise to a solution of 1-bromo-4-methoxynaphthalene **S1** (1.19 g, 5.00 mmol), NEt_3_ (2.79 mL, 20 mmol) and Pd(ddpf)Cl_2_·CH_2_Cl_2_ (510 mg, 0.625 mmol) in anhydrous dioxane (15 mL) and the reaction mixture heated at 100 °C for 23 h. After cooling to room temperature, the reaction was quenched cautiously with water (~20 mL) and extracted with CH_2_Cl_2_ (3×35 mL). The combined organic extracts were washed with brine (50 mL), dried (Na_2_SO_4_) and concentrated under reduced pressure. Purification by flash chromatography (SiO_2_; EtOAc/heptane; 0-30%) afforded the pinacol ester as an off-white solid (1.09 g, 77%). ^1^H NMR (400 MHz, CDCl_3_) δ = 1.41 (s, 12H), 4.02 (s, 3H), 6.82 (d, *J* = 7.8 Hz, 1H), 7.43-7.48 (m, 1H), 7.53-7.57 (m, 1H), 8.04 (d, *J* = 7.8 Hz, 1H), 8.28 (dd, *J* = 0.9, 8.3 Hz, 1H), 8.75 (d, *J* = 8.3 Hz, 1H). ^13^C NMR (100 MHz, CDCl_3_) δ = 25.1, 55.6, 83.5, 103.3, 122.1, 125.0, 125.5, 127.0, 128.3, 137.0, 138.3, 158.3. HRMS (ESI+) (m/z): [M+H]^+^ calcd. for C_17_H_22_BO_3_, 285.1657; found, 285.1643.

**Methyl (3*R*)-7-[(4-methoxynaphthalen-1-yl)methyl]-8-cyclopropyl-5-oxo-2,3-dihydro-5*H*-[1,3]thiazolo[3,2-*a*]pyridine-3-carboxylate (S4).** The chloromethyl analogue **S3** (150 mg, 0.500 mmol), boronic acid pinacol ester **S2** (284.2 mg, 1.00 mmol), PdCl_2_(PPh_3_)_2_ (35 mg, 0.005 mmol) and KF (58 mg, 1.00 mmol) were dissolved in anhydrous MeOH (8 mL) and heated by MWI at 110 °C for 10 min. The reaction mixture was quenched with saturated aqueous NaHCO_3_ solution (15 mL) and extracted with CH_2_Cl_2_ (3×20 mL). The combined organic extracts were washed successively with water and brine (50 mL each), dried (Na_2_SO_4_) and concentrated under reduced pressure. The crude residue was dissolved in EtOAc and filtered through Celite^®^, washing with EtOAc, and again concentrated under reduced pressure. Purification by flash chromatography (SiO_2_; EtOAc/heptane; 20-100%) afforded the product as a pale brown oil (151 mg, 72%). ^1^H NMR (400 MHz, CDCl_3_) δ = 0.69-0.78 (m, 2H), 0.87-1.03 (m, 2H), 1.61-1.71 (m, 1H), 3.50 (dd, *J* = 2.3, 11.8 Hz, 1H), 3.66 (dd, *J =* 8.5, 11.7 Hz, 1H), 3.78 (s, 3H), 4.00 (s, 3H), 4.25 (d, *J* = 17.3 Hz, 1H), 4.41 (d, *J* = 17.3 Hz, 1H), 5.56 (dd, *J =* 2.2, 8.5 Hz, 1H), 5.75 (s, 1H), 6.75 (d, *J* = 7.8 Hz, 1H), 7.17 (d, *J* = 7.8 Hz, 1H), 7.44-7.50 (m, 2H), 7.68-7.74 (m, 1H), 8.28-8.33 (m, 1H). ^13^C NMR (100 MHz, CDCl_3_) δ = 7.6, 7.9, 11.3, 31.9, 36.0, 53.4, 55.7, 62.8, 103.5, 113.9, 115.4, 122.9, 123.8, 125.2, 125.9, 126.2, 126.8, 127.7, 132.8, 146.9, 155.1, 157.5, 161.4, 168.8. HRMS (ESI+) (m/z): [M+H]^+^ calcd. for C_24_H_24_NO_4_S, 422.1421; found, 422.1411.

**(3*R*)-7-[(4-methoxynaphthalen-1-yl)methyl]-8-cyclopropyl-5-oxo-2,3-dihydro-5*H*-[1,3]thiazolo[3,2-*a*]pyridine-3-carboxylate (KSK85).**

The title compound was prepared by adaptation of a reported procedure (1). Lithium hydroxide (1.0 M aqueous solution, 0.380 mL, 0.380 mmol) was added to a cooled solution (0 ºC) of the methyl ester **S4** (80 mg, 0.190 mmol) in THF (8 mL) and stirred at room temperature overnight. The reaction was acidified (circa pH 1) with aqueous HCl (1.0 M), extracted with EtOAc, dried (Na_2_SO_4_) and the solvent removed under reduced pressure. Purification by flash chromatography (SiO_2_, MeOH/CH_2_Cl_2_/AcOH, 95:5:1 to MeOH/CH_2_Cl_2_, 97:3) and subsequent freeze-drying (H_2_O:MeCN; ~ 3:1) afforded the product as a white solid (62 mg, 80%). [α]_D_^20^ = -5.2 (c = 0.6, CHCl_3_:MeOH; 9:1). ^1^H NMR (400 MHz, DMSO-d_6_) δ = 0.60-0.69 (m, 1H), 0.72-0.79 (m, 1H), 0.85-0.98 (m, 2H), 1-69-1.77 (m, 1H), 3.50 (dd, *J* = 1.8, 11.9 Hz, 1H), 3.79 (dd, *J =* 9.0, 11.9 Hz, 1H), 3.98 (s, 3H), 4.30 (d, *J* = 17.5 Hz, 1H), 4.39 (d, *J* = 17.4 Hz, 1H), 5.23 (s, 1H), 5.32 (dd, *J =* 1.8, 9.1 Hz, 1H), 6.97 (d, *J* = 8.0 Hz, 1H), 7.30 (d, *J* = 7.9 Hz, 1H), 7.50-7.57 (m, 2H), 7.77-7.80 (m, 1H), 8.20-8.24 (m, 1H), 13.36 (br s, 1H). ^13^C NMR (100 MHz, DMSO-d_6_) δ = 7.7, 7.9, 11.2, 31.9, 35.4, 56.0, 63.1, 104.5, 112.3, 113.8, 122.6, 124.6, 125.6, 125.7, 126.6, 127.3, 128.3, 132.8, 148.4, 154.5, 157.1, 160.4, 170.2. HRMS (ESI+) (m/z): [M+ Na]^+^ calcd. for C_23_H_21_NNaO_4_S, 430.1089; found, 430.1071.

**(3*R*)-9-cyclopropyl-5-oxo-7-phenyl-2,3,5,7-tetrahydropyrrolo[3,4-*d*][1,3]thiazolo[3,2-*a*]pyridine-3-carboxylic acid (GKP42)** The title compound was prepared by adaptation of a reported procedure (1). Lithium hydroxide (1.0 M aqueous solution, 0.606 mL, 0.606 mmol) was added to a cooled solution (0 ºC) of the methyl ester **S5** (111 mg, 0.303 mmol) in MeOH/THF (1:2; 6 mL) and stirred at room temperature for 48 h, after which additional lithium hydroxide (1.0 M aqueous solution, 0.606 mL, 0.606 mmol) was added and the reaction stirred for a further 8 h. The reaction was cooled to 0 ºC, acidified with aqueous HCl (0.1 M, 20 mL), extracted with 5% MeOH in CH_2_Cl_2_ (4 × 25 mL) and the solvent removed under reduced pressure. Purification by HPLC (mobile phase: MeCN/H_2_O with 0.75% formic acid each, 35-100% for 40 min; *t*_R_ = 21.48 min) and subsequent freeze-drying (H_2_O:MeCN; ~ 3:1) gave the product as a pale orange solid (38 mg, 67%). [α]_D_^20^ = -35.8 (*c* = 0.6, DMSO-*d_6_*). ^1^H NMR (400 MHz, DMSO-*d_6_*) δ = 0.63-0.77 (m, 2H), 0.83-0.93 (m, 2H), 1.65-1.74 (m, 1H), 3.48 (dd, *J* = 1.2, 11.7 Hz, 1H), 3.74 (dd, *J* = 8.2, 11.7 Hz, 1H), 5.42 (dd, *J* = 1.2, 8.2 Hz, 1H), 7.36-7.41 (m, 1H), 7.47 (d, *J* = 2.3 Hz, 1H), 7.50-7.56 (m, 2H), 7.78-7.81 (m, 2H), 8.09 (d, *J* = 2.3 Hz, 1H). ^13^C NMR (100 MHz, DMSO-*d_6_*) δ = 5.6, 5.9, 10.9, 31.4, 60.9, 104.7, 109.7, 115.7, 118.3, 120.9, 127.1, 127.2, 129.8, 134.8, 139.3, 157.9, 170.5. HRMS (ESI+) (*m/z*): [M+H]^+^ calcd. for C_19_H_17_N_2_O_3_S, 353.0954; found, 353.0925.

**Supplemental Synthetic Schemes**

**Scheme S1 – Synthesis of KSK85**

**Scheme S2 - Synthesis of GKP42**

**SUPPLEMENTAL STRUCTURE-ACTIVITY RELATIONSHIP ANALYSIS**

The subset of small molecules employed in screening provided initial information on the structure-activity relationship underlying attenuation of *cag* T4SS function (Table S1). The two active compounds were highly structurally similar, incorporating a central bicyclic ring-fused thiazolino 2-pyridone, a C-3 carboxylic acid, a C-8 cyclopropyl substituent and a C-7 CH2-(1-naphthyl) which varied only through the methoxy substituent present in KSK85. Introducing a benzylic substituent into the C-2 position on the thiazolo ring, an important feature for pilicide activity in *E. coli,* was not tolerated (**EC240**, Table S1) (1, 4). Similarly, increasing the size of the C-8 substituent on the 2-pyridone ring was not tolerated (**PhenylC8, FN075**) (5). Appending amino moieties onto the C-6 position abolished activity, demonstrated by **MS68** and **NP048**, while fusing the C-6 and C-7 positions ablated activity, with both the tricyclic heterocyclic analogues **MS218** and **GKP42** inactive. These preliminary data provide invaluable information to guide the design of improved inhibitors of T4SS related processes.

**Supplemental MATERIALS AND Methods**

**IL-8 induction assays.** *H. pylori* was harvested from blood agar plates after 48 h of growth, re-suspended in complete RPMI at approximately 10^9^ CFUs/ml and incubated for 1 h with shaking in the presence of the indicated compounds or DMSO at a concentration of 150 μM. The RPMI medium was removed from AGS cell monolayers and was replaced with an equivalent volume of complete RPMI supplemented with compound (ranging from 150 μM to 25 μM), or equivalent volume of DMSO vehicle alone immediately prior to inoculation with *H. pylori*. AGS cell monolayers (80-90% confluent) were infected at a multiplicity of infection (MOI) of 100:1. Triplicate wells per condition were inoculated. Following 4 h incubation with *H. pylori*, AGS cell supernatants were collected, and IL-8 levels were quantified by anti-human IL-8 ELISA (R&D). Levels of IL-8 secreted by AGS cells in response to *H. pylori* infection in the presence of individual compounds were compared to the levels of IL-8 secreted in the DMSO control wells (set to 100% in all experiments).

**CagA translocation assay.** Translocation of CagA into AGS cells was analyzed as described in (6). Briefly, *H. pylori* was pre-treated with compounds and co-cultured with AGS cells at an MOI of 100 in triplicate in complete RPMI supplemented with 150 μM compound or DMSO for 4.5 h at 37 °C in 5% CO_2_ atmosphere. AGS monolayers were washed in PBS containing 2 mM sodium orthovanadate to remove non-adherent bacteria, and AGS cells were lysed in 1% NP-40 buffer containing Complete Mini (EDTA-free) Protease Inhibitor (Roche) and PhosSTOP pan phosphatase inhibitor (Roche). CagA translocation was assessed by separation of the soluble cellular fraction by SDS-PAGE and immunoblot analysis using anti-phosphotyrosine antibody (α-PY99, Santa Cruz). Levels of phospho-CagA were normalized to levels of total CagA (α-CagA, Santa Cruz) in 5 independent experiments. Densitometry analysis was performed in ImageJ.

**Bacterial adherence to gastric epithelial cells.** Compound pre-treated *H. pylori* were added to AGS cells in the presence of 150 μM compound and treated as described for IL-8 induction studies. After 4 hours of co-culture, RPMI was aspirated and the wells were washed 5 times with PBS. Adherent bacteria and cell monolayers were fixed in 4% paraformaldehyde at room temperature for 15 min, followed by blocking in 1% BSA in PBS containing 0.1% Triton X-100 for 1 h at room temperature. Bacteria were stained with antisera raised against soluble *H. pylori* proteins (α-*H. pylori*) at a dilution of 1:1000 in 1% BSA for 1 h at room temperature, followed by 5 washes in PBS and incubation in goat anti-rabbit secondary antibody conjugated to Alexa-488 (1:5000) (Life Technologies). Wells were washed 3 times in PBS and fluorescence was measured using a BioTek Synergy 4 plate reader. As a control, AGS cell monolayers were stained with primary and secondary antibody or secondary antibody alone. Average fluorescence of triplicate wells was normalized to control wells, and *H. pylori* adherence to AGS cells in the presence of 150 μM compound was compared to *H. pylori* adherence in the presence of DMSO vehicle alone.

**VacA secretion assay.** WT *H. pylori* was cultured overnight in brucella broth containing 5% FBS supplemented with either 100 μM DMSO, C10, KSK85, or GKP42 as previously described (7). Bacterial cultures were centrifuged (2,000 x g) to separate bacterial cells and cell culture supernatants. Bacterial pellets were re-suspended in PBS at a final volume equivalent to the volume of the supernatant. Equal volumes of each re-suspended cell pellet and its corresponding cell culture supernatant were added to 2X SDS buffer (Bio-Rad), and equal volumes of each sample were resolved by SDS-PAGE (Bio-Rad). Immunoblotting to probe for secreted (supernatant) and bacterial cell-associated VacA (pellet) was performed as previously described (7).

**Field emission gun scanning electron microscopy.** Samples were fixed with 2.0% paraformaldehyde, 2.5% glutaraldehyde in 0.05 M sodium cacodylate buffer, and secondarily fixed with 0.1% osmium tetroxide. Sequential dehydration was performed by washing with increasing concentrations of ethanol. Samples were dried at the critical point with a Tousimis critical point dryer. Coverslips were mounted onto aluminum stubs and sputter coated with gold-palladium and imaged with an FEI Quanta 250 FEG-SEM.

**Agroinfiltration and GUS Assay.** To determine the efficacy of test compounds to prevent transfer of T-DNA from a Ti plasmid harbored by *Agrobacterium tumefaciens* to the nuclear DNA of a plant host cell, *A. tumefaciens* (GV3101) with and without a suitable expression vector (pCAMBIA 1305.2) was utilized. The pCAMBIA 1305.2 expression vector contains an intron-containing β-glucuronidase (GUS) gene inserted downstream of the CaMV 35S promoter (a strong, constitutive gene expression promoter in plants) into the T-DNA region of the plasmid (www.cambia.org). The pCAMBIA 1305.2 vector was transformed into *A. tumefaciens* GV3101 via freeze-thaw method (8), and recombinant GV3101 lines were selected for kanamycin resistance. *A. tumefaciens* with the pCAMBIA 1305.2 expression vector were subsequently grown in LB liquid growth media containing 100 µg/ml of kanamycin and 10 µg/ml of rifampicin, while control *A. tumefaciens* not harboring the plasmid vector were grown in LB media containing only 10 µg/ml of rifampicin. *A. tumefaciens* cultures were grown overnight at 28 °C to an OD_600_ of 0.8. The cells were then pelleted by centrifugation (2000 x g for 10 min), washed twice with water to remove residual LB, and re-suspended in infiltration buffer (10 mM MES pH 5.7, 10 mM MgCl_2_) at an OD_600_ of 0.8. Aliquots of the cell cultures were then incubated with test compounds at 50, 150, or 450 µM concentrations for 1 hour prior to leaf infiltration. As a vehicle control, *A. tumefaciens* cultures harboring the pCAMBIA expression vector were incubated in equivalent concentrations of DMSO, and DNA transfer-deficient negative controls of *A. tumefaciens* not harboring the Ti-expression vector were treated identically.

Agroinfiltration of homogenous bacterial cultures containing the indicated final concentration of ring-fused 2-pyridone compound was performed on 2-month old *Nicotiana benthamiana* plants using expanding leaves of approximately 6 cm long by 5 cm wide. Six zones (two zones for controls and four zones for each experimental condition) were infiltrated per leaf with approximately 100 µl of compound-treated *A. tumefaciens* suspensions infiltrated per zone. Agroinfiltration was performed using a 1 ml needleless syringe to gently inject bacterial suspensions into the plant interstitial space from the abaxial surface. The infiltration zones were visible as water soaked areas that were subsequently demarcated on upper side of each leaf. Infiltration buffer within each inoculation zone was absorbed within hours following injection; therefore, the infiltration zones were injected twice more at 12 and 24 h post-initial infiltration with infiltration buffer containing the respective test compounds at the appropriate concentrations.

Leaves containing Agroinfiltration zones were harvested after 48 h of incubation, the empirically derived time point at which T-DNA transfer had occurred for sufficient detection of β-glucuronidase (GUS) enzyme activity. GUS activity was initially assessed using X-gluc (5-bromo-4-chloro-3-indolyl-β-D-glucuronide cyclohexylammonium salt) (Goldbiotech) as a substrate in GUS staining buffer (100 mM sodium phosphate pH 7.0, 10 mM EDTA, 0.5 mM potassium ferro-cyanide, 0.5 mM potassium ferricyanide, 0.05% Triton-X-100, 2 mM X-gluc). The quantitative GUS fluorometric assay was used to measure GUS gene expression (9). Briefly, leaf discs were homogenized in sodium phosphate buffer, and homogenates were separated by centrifugation at 10,000 x g. Total protein concentration of each cleared homogenate supernatant was determined by Bradford reagent (Bio-Rad) using bovine serum albumin (BSA) to generate a standard curve. Normalized supernatants were assayed for β-glucuronidase activity by measuring the GUS cleavage-dependent conversion of methylumbelliferyl-β-D-glucuronide (MUG) substrate to fluorescent 4-methylumbelliferone (MU). GUS activity was expressed as nanomoles of MU produced per minute per milligram of protein. Average GUS activity from at least 3 independent biological replicate plants (minimum of three leaves per plant) is expressed as a percent (%) of the DMSO vehicle control (values set to 100%) ± SEM; statistical significance for each compound treated condition was established by one-way ANOVA.

**Supplemental References**

1. **Chorell E, Pinkner JS, Phan G, Edvinsson S, Buelens F, Remaut H, Waksman G, Hultgren SJ, Almqvist F.** 2010. Design and synthesis of C-2 substituted thiazolo and dihydrothiazolo ring-fused 2-pyridones: pilicides with increased antivirulence activity. Journal of Medicinal Chemistry **53:**5690-5695.

2. **Sellstedt M, Prasad GK, Krishnan SK, Almqvist F.** 2012. Directed diversity-oriented synthesis. Ring-fused 5- to 10-membered rings from a common peptidomimetic 2-pyridone precursor. Tetrahedron Letters **53:**6022-6024.

3. **Carreno MC, Garcia Ruano JL, Sanz G, Toledo MA, Urbano A.** 1995. N-bromosuccinimide in acetonitrile: A mild and regiospecific nuclear brominating reagent for methoxybenzenes and naphthalenes. The Journal of Organic Chemistry **60:**5328-5331.

4. **Greene SE, Pinkner JS, Chorell E, Dodson KW, Shaffer CL, Conover MS, Livny J, Hadjifrangiskou M, Almqvist F, Hultgren SJ.** 2014. Pilicide ec240 disrupts virulence circuits in uropathogenic *Escherichia coli*. mBio **5**.

5. **Cegelski L, Pinkner JS, Hammer ND, Cusumano CK, Hung CS, Chorell E, Åberg V, Walker JN, Seed PC, Almqvist F, Chapman MR, Hultgren SJ.** 2009. Small-molecule inhibitors target *Escherichia coli* amyloid biogenesis and biofilm formation. Nat Chem Biol **5:**913-919.

6. **Shaffer CL, Gaddy JA, Loh JT, Johnson EM, Hill S, Hennig EE, McClain MS, McDonald WH, Cover TL.** 2011. *Helicobacter pylori* exploits a unique repertoire of type IV secretion system components for pilus assembly at the bacteria-host cell interface. PLoS Pathog **7:**e1002237.

7. **Gonzalez-Rivera C, Algood HM, Radin JN, McClain MS, Cover TL.** 2012. The intermediate region of *Helicobacter pylori* VacA is a determinant of toxin potency in a Jurkat T cell assay. Infect Immun **80:**2578-2588.

8. **Holsters M, de Waele D, Depicker A, Messens E, van Montagu M, Schell J.** 1978. Transfection and transformation of *Agrobacterium tumefaciens*. Molecular and General Genetics MGG **163:**181-187.

9. **Jefferson RA, Kavanagh TA, Bevan MW.** 1987. GUS fusions: beta-glucuronidase as a sensitive and versatile gene fusion marker in higher plants. Embo J **6:**3901-3907.
