# Supplementary figures and images for: Peptidomimetic Small Molecules Disrupt Type IV Secretion System Activity in Diverse Bacterial Pathogens
Source: mBio. 2016 Apr 26;7(2):e00221-16. doi: 10.1128/mBio.00221-16 (PMC4850256; doi:10.1128/mBio.00221-16)

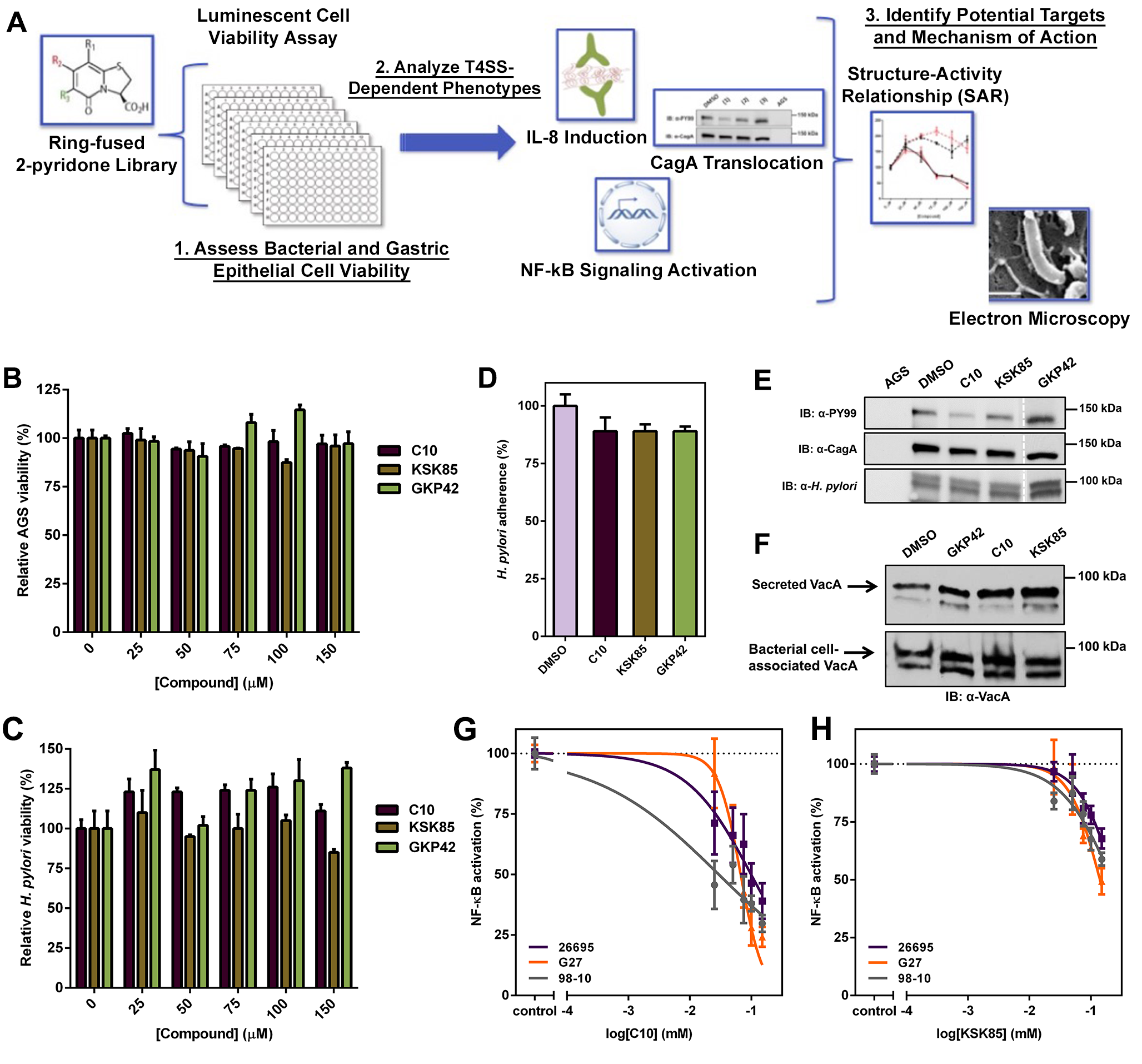

Supplement: Figure S1 — Effects of peptidomimetic small molecules on cell viability and T4SS-independent processes. (A) Schematic depicting the workflow of compound screening. A collection of ring-fused 2-pyridone compounds was initially evaluated at final concentrations of 150 µM for impact on host cell or H. pylori viability. Compounds that did not negatively affect either host or bacterial cell viability were subsequently evaluated for inhibition of H. pylori cag T4SS-dependent processes, including disruption of CagA translocation, induction of IL-8 secretion by cultured gastric epithelial cells, and NF-κB activation. (B and C) AGS (B) and H. pylori (C) cell viability as determined by the level of cellular ATP content after 6 h exposure to compounds. (D) Total H. pylori adherence to gastric epithelial cell monolayers in the presence of ring-fused 2-pyridones (final concentrations of 150 µM for all assayed compounds).Values in panels B to D represent the means plus SEM (error bars) of at least three biological replicates. (E) Representative image depicting CagA translocation into cultured gastric epithelial cells (tyrosine-phosphorylated CagA, immunoblotting with anti-PY99 antibody [IB: α-PY99]) versus levels of total CagA (IB: α-CagA). (F) Immunoblot depicting the relative amounts of H. pylori VacA secreted into cell culture supernatants when bacteria were grown in the presence of compounds or DMSO. (G and H) Effects of C10 (G) and KSK85 (H) on cag T4SS-dependent activation of NF-κB in an AGS reporter cell line by multiple H. pylori strains. Data points in panels G and H depict the means ± SEM (error bars) of at least three biological replicate experiments. Download [file mbo002162780sf1.tif]

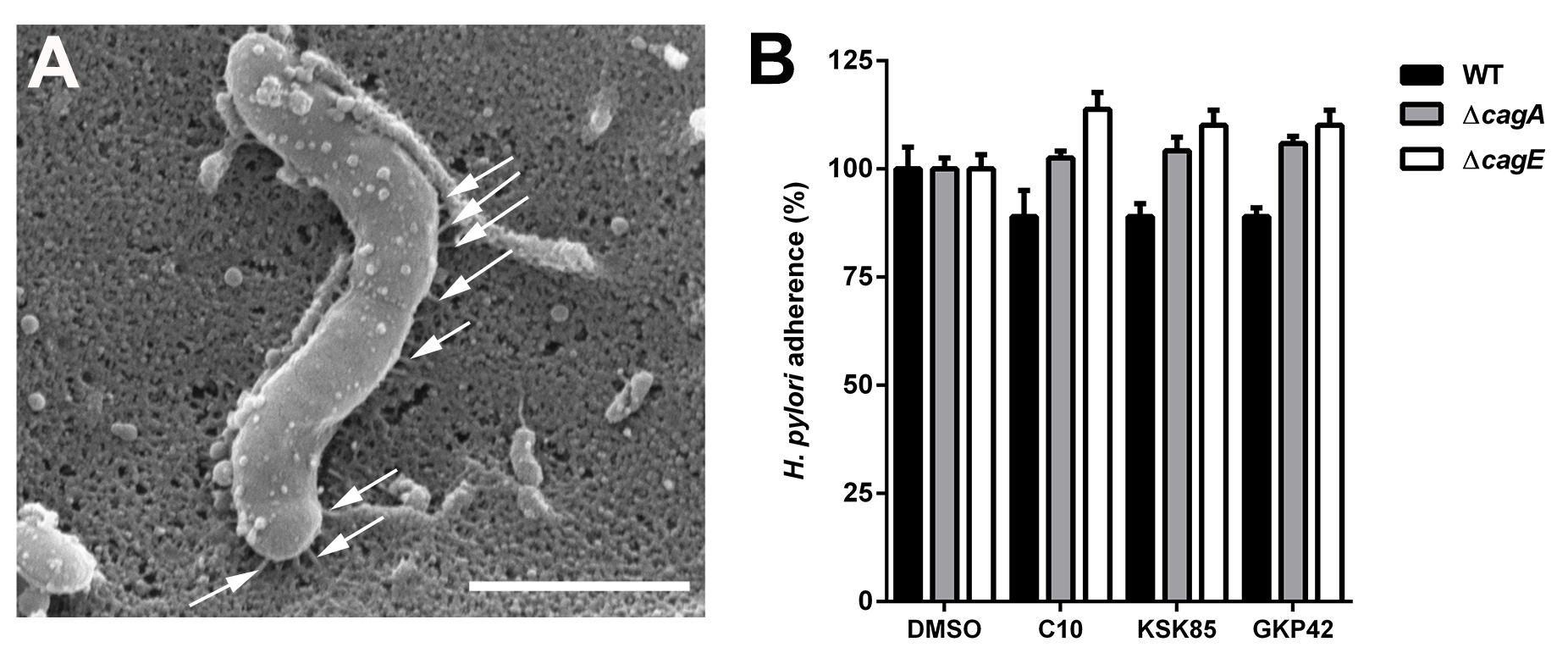

Supplement: Figure S2 — CagA is not required for T4SS pilus production or adherence to gastric epithelial cells. (A) Scanning electron microscopy analysis of T4SS pilus assembly by H. pylori ΔcagA mutant. Bar, 1 µm. (B) Total bacterial adherence of WT, ΔcagA, and ΔcagE strains to gastric epithelial cells in the presence of compounds. Bars represent the adherence of each strain normalized to DMSO-treated samples (mean plus standard error) and are representative of at least two biological replicate experiments. Download [file mbo002162780sf2.tif]

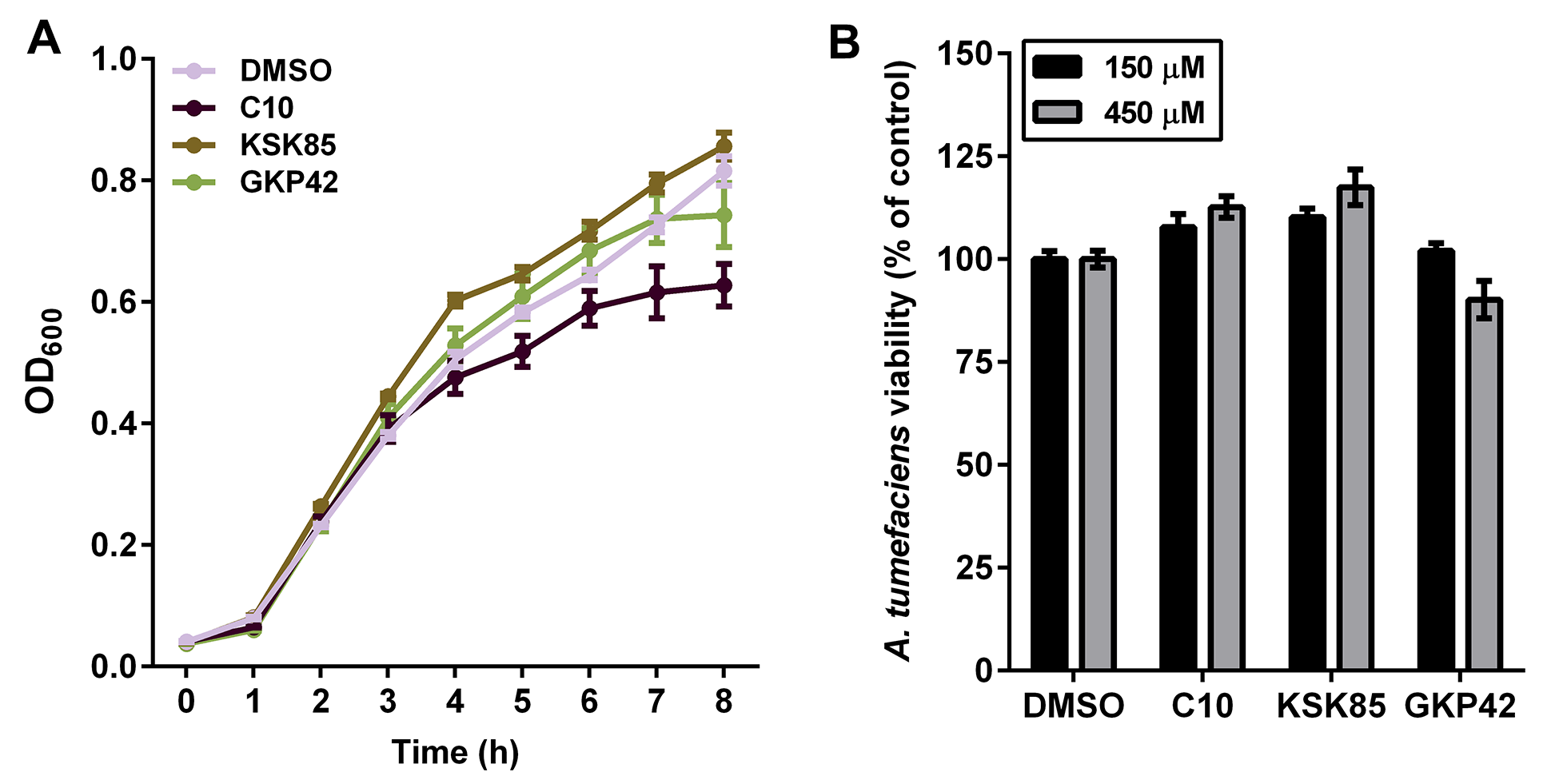

Supplement: Figure S3 — Effects of peptidomimetic compounds on E. coli and A. tumefaciens viability. (A) Optical density measurements of the growth of E. coli MG1655 harboring pKM101 in the presence of vehicle, C10, KSK85, or GKP42 measured at 1 h intervals. (B) A. tumefaciens cell viability as determined by the level of cellular ATP content after 24 h growth in the presence of compounds at the indicated concentrations. Data points in panel A represent the mean OD600 of six independent samples. Data in panel B represent the mean cellular ATP content ± SEM compared to DMSO vehicle control samples and are representative of two biological replicate experiments. Download [file mbo002162780sf3.tif]

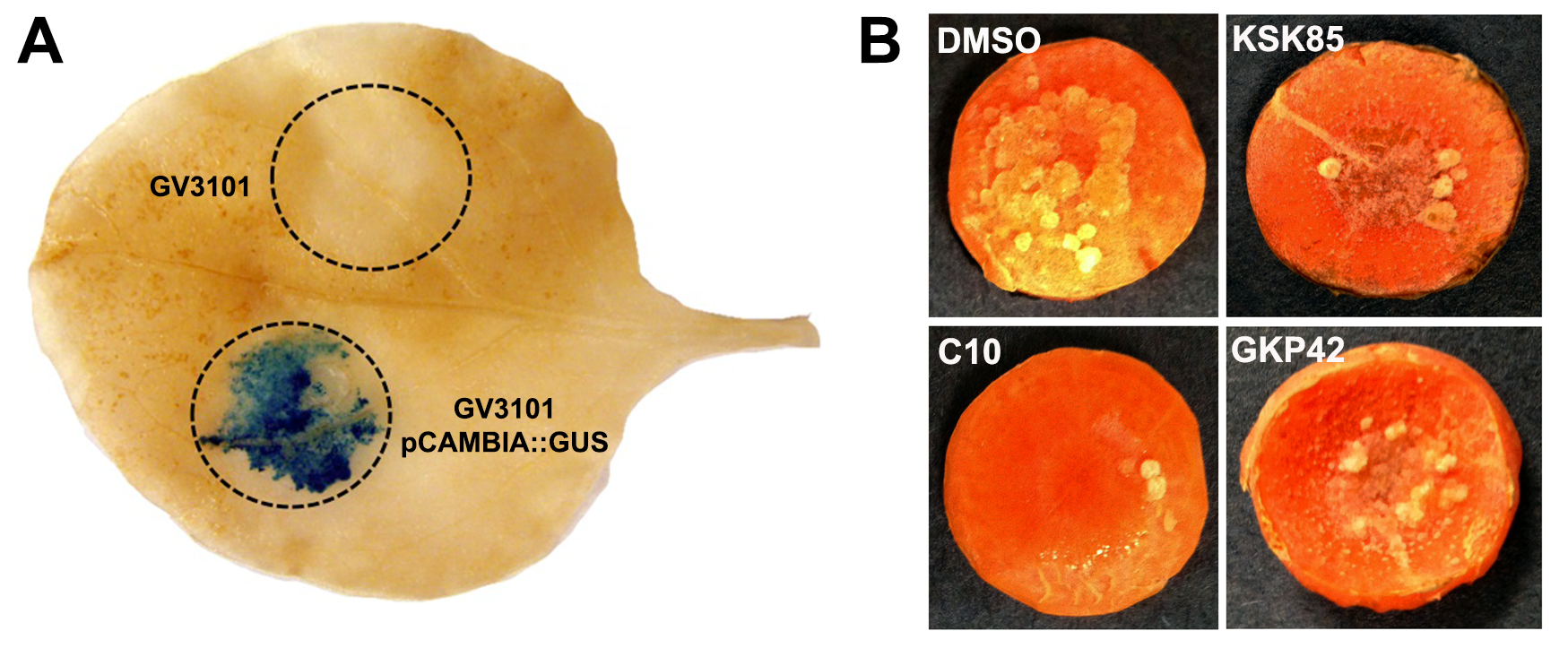

Supplement: Figure S4 — Qualitative assessment of A. tumefaciens vir T4SS-dependent phenotypes. (A) Qualitative representation of T-DNA incorporation and GUS expression in tobacco leaves. Young, expanding leaves of Nicotiana benthamiana were infiltrated with A. tumefaciens GV3101 or A. tumefaciens GV3101 harboring pCAMBIA 1305.2 intronic GUS reporter gene expression cassette (pCAMBIA::GUS). A representative image of an N. benthamiana leaf stained histochemically for GUS enzyme activity demonstrating negative (GV3101) and positive (GV3101 pCAMBIA::GUS) incorporation of the β-glucuronidase reporter gene into N. benthamiana nuclear DNA is shown. (B) Qualitative carrot disk tumor assay demonstrating marked reduction of A. tumefaciens C58-induced tumors after a single administration (150 µM) of compound or equivalent volume of DMSO. Download [file mbo002162780sf4.tif]
