# Supplementary material for: Peptidomimetic Small Molecules Disrupt Type IV Secretion System Activity in Diverse Bacterial Pathogens
Source: mBio. 2016 Apr 26;7(2):e00221-16. doi: 10.1128/mBio.00221-16 (PMC4850256; doi:10.1128/mBio.00221-16)
Supplement: Table S1 — Composition of peptidomimetic 2-pyridone focused screening library. The compounds (150 µM) were screened for phenotypic disruption of cag T4SS activity as measured by a significant decrease in IL-8 secretion or a significant decrease in NF-κB activation induced by WT H. pylori in co-culture with AGS gastric epithelial cells. The compounds were determined to be toxic to either AGS cells or H. pylori by a significant decrease in cellular ATP at 18 h of incubation. [file mbo002162780st1.docx]

**Table S1. Composition of peptidomimetic 2-pyridone focused screening library^a^**

| **Compound** (Reference) | **AGS**  **Toxicity^b^** | ***H. pylori***  **Toxicity^b^** | ***cag* T4SS**  **inhibition** | **Compound** (Reference) | **AGS**  **Toxicity^b^** | ***H. pylori***  **Toxicity^b^** | ***cag* T4SS**  **inhibition** |
| --- | --- | --- | --- | --- | --- | --- | --- |
|   **C10** (8) | **–** | **–** | **+++** |   **GKP42** (2) | **–** | **–** | **–** |
|   **CB151** (9) | **–** | **Toxic** | **–** |   **KSK85**  This study | **–** | **–** | **+++** |
|   **CB160** (9) | **–** | **Toxic** | **–** |   **MS218** (10) | **–** | **–** | **–** |
|   **CB220** (11) | **–** | **–** | **–** |   **MS383** (11) | **–** | **–** | **–** |
|   **CB223** (11) | **–** | **–** | **–** |   **MS400** (11) | **–** | **–** | **–** |
|   **EC016** (12) | **–** | **–** | **–** |   **MS542** (13) | **–** | **–** | **–** |
|   **EC240** (1) | **–** | **Toxic** | **–** |   **MS610** (2) | **–** | **–** | **–** |
|   **EC312** (9) | **–** | **–** | **–** |   **NP047** (14) | **–** | **–** | **–** |
|   **EC341** (9) | **–** | **Toxic** | **–** |   **NP048** (14) | **–** | **–** | **–** |
|   **EC369** (9) | **Toxic** | **–** | **–** |   **NP154** (15) | **–** | **–** | **–** |
|   **FN075** (12) | **–** | **Toxic** | **–** |   **PhenylC8** (8) | **–** | **–** | **–** |

^a^Compounds (150 µM) were screened for phenotypic disruption of *cag* T4SS activity as measured by a significant decrease in IL-8 secretion or a significant decrease in NF-kB activation induced by WT *H. pylori* in co-culture with AGS gastric epithelial cells.

^b^Compounds were determined to be toxic to either AGS cells or *H. pylori* by a significant decrease in cellular ATP at 18 h of incubation.
